# *Domain Auto Finder* (*DAFi*) program: the analysis of single-crystal X-ray diffraction data from polycrystalline samples

**DOI:** 10.1107/S1600576722008081

**Published:** 2022-09-28

**Authors:** Andrey Aslandukov, Matvii Aslandukov, Natalia Dubrovinskaia, Leonid Dubrovinsky

**Affiliations:** a Material Physics and Technology at Extreme Conditions, Laboratory of Crystallography, University of Bayreuth, Universitaetsstrasse 30, Bayreuth, D-95440, Germany; b Bayerisches Geoinstitut, University of Bayreuth, Bayreuth, D-95440, Germany; c Kharkiv National University of Radio Electronics, Nauky Avenue 14, Kharkiv, 61166, Ukraine; d Department of Physics, Chemistry and Biology (IFM), Linkoping University, Linköping, SE 581 83, Sweden; HPSTAR and Harbin Institute of Technology, People’s Republic of China

**Keywords:** single-crystal domain auto finder, *DAFi*, single-crystal X-ray diffraction, polycrystalline samples, multiphase mixtures

## Abstract

This paper presents the *Domain Auto Finder* (*DAFi*) program and its application to the analysis of single-crystal X-ray diffraction (SC-XRD) data from multiphase mixtures of microcrystalline solids and powders. Superposition of numerous reflections originating from a large number of single-crystal domains of the same and/or different (especially unknown) phases usually precludes the sorting of reflections coming from individual domains, making their automatic indexing impossible. The *DAFi* algorithm is designed to quickly find subsets of reflections from individual domains in a whole set of SC-XRD data. Further indexing of all found subsets can be easily performed using widely accessible crystallographic packages. As the algorithm neither requires *a priori* crystallographic information nor is limited by the number of phases or individual domains, *DAFi* is powerful software to be used for studies of multiphase polycrystalline and microcrystalline (powder) materials. The algorithm is validated by testing on X-ray diffraction data sets obtained from real samples: a multi-mineral basalt rock at ambient conditions and products of the chemical reaction of yttrium and nitro­gen in a laser-heated diamond anvil cell at 50 GPa. The high performance of the *DAFi* algorithm means it can be used for processing SC-XRD data online during experiments at synchrotron facilities.

## Abstract 2

This paper presents the *Domain Auto Finder* (*DAFi*) program and its application to the analysis of single-crystal X-ray diffraction (SC-XRD) data from multiphase mixtures of microcrystalline solids and powders. The *DAFi* algorithm is designed to quickly find subsets of reflections from individual domains in a whole set of SC-XRD data and neither requires *a priori* crystallographic information nor is limited by the number of phases or individual domains.

## 1. Introduction

For more than a century, single-crystal X-ray diffraction (SC-XRD) has been a powerful method for determining the structure of crystalline solids. Until very recently it could be applied to single crystals not smaller than dozens of micrometres, but many compounds are only available in a polycrystalline form or as fine powders. State-of-the-art powder X-ray diffraction (XRD) data analysis, including Rietveld refinement in combination with *ab initio* structure search, can help with structure interpretation if sufficiently large crystals are unavailable and their preparation or growth is infeasible. This concerns investigations of natural objects or drugs, *in situ* studies of matter under extreme conditions, or processes in solids involving domain formation or reconstructive phase transitions. However, when it comes to multiphase systems with unknown microcrystalline compounds, the problem of structure solution for individual components becomes even more difficult.

In recent decades, the development of third- and fourth-generation synchrotrons, such as the Advanced Photon Source (in Lemont, USA), PETRA III (in Hamburg, Germany) and the ESRF (in Grenoble, France), with the ESRF–EBS (the Extremely Brilliant Source, the ESRF’s facility upgrade over 2015–2022, which increases the brilliance and coherence of the X-ray beams produced by a factor of 100 compared with present-day light sources; https://www.esrf.fr/about/upgrade), has provided users with new opportunities. At the cutting-edge beamlines, such as ID11 at the ESRF, the size of the X-ray beam (0.5 × 0.5 µm FWHM) is commensurate with the size of crystalline domains of polycrystalline samples or fine powder particles, which makes it possible to study each micrometre- to submicrometre-size grain individually by methods of SC-XRD, considering the sphere of confusion of the diffractometer of only a few hundred nanometres. This approach was devised for and first applied to studying products of chemical reactions and phase transformations in laser-heated diamond anvil cells (DACs); this has led to discoveries of many exotic compounds, revealing their crystal structures *in situ* under high pressure (*e.g.* Bykova *et al.*, 2016 ▸, 2018 ▸; Laniel, Winkler, Bykova *et al.*, 2020 ▸; Laniel, Winkler, Fedotenko *et al.*, 2020 ▸; Bykov *et al.*, 2020 ▸, 2021 ▸; Aslandukova *et al.*, 2021 ▸; Dubrovinskaia & Dubrovinsky, 2018 ▸; Ceppatelli *et al.*, 2022 ▸).

Still, processing SC-XRD data containing a lot of reflections coming from numerous crystalline grains is a difficult task, especially in the presence of a few different phases in a multicomponent system and/or in the absence of any *a priori* information about their chemical composition and/or basic crystallographic characteristics, such as the unit-cell parameters. The diffraction data collected from samples under high pressure in a DAC are additionally complicated by undesired but unavoidable reflections from diamond anvils, pressure-transmitting media, gasket materials and other factors. Therefore, the development of software which would allow an automatic separation of the reflections originating from an individual crystalline domain, *i.e.* a search for the domain in a complex pattern of spots in the reciprocal space, is an urgent task aimed at rationalizing SC-XRD data processing and making it routine for inexperienced users.

To date, several programs have been developed for multigrain indexing. If the unit-cell parameters are known *a priori*, *e.g.* from powder XRD data, indexing means finding the orientation matrices of the grains in the sample and sorting the reciprocal-space vectors with regard to the grain of origin. Following the presentation of the program *GRAINDEX* (Lauridsen *et al.*, 2001 ▸), several alternative approaches have been proposed (Wright, 2006 ▸; Ludwig *et al.*, 2009 ▸; Moscicki *et al.*, 2009 ▸; Schmidt, 2014 ▸). The programs *ImageD11* (Wright, 2006 ▸) and *GrainSpotter* (Schmidt, 2014 ▸) are now incorporated into the *FABLE* (*Fully Automatic BeamLine Experiments*) package (Sørensen *et al.*, 2012 ▸). The main limitation of the above-mentioned software is that it is designed to be applied almost exclusively to the analysis of mono-phase materials. Furthermore, the multigrain indexing programs mentioned above all assume that the space group (or at least symmetry) and the unit-cell parameters of phases are known. One straightforward way to generalize the previous approaches is to apply the multigrain indexing algorithms repeatedly, once for each phase (Jimenez-Melero *et al.*, 2011 ▸; Sørensen *et al.*, 2012 ▸), but this still requires the phases to be identified in advance.

To our knowledge, there have only been a few proposals for dealing with unknown phases, based on a fast Fourier transform approach (Sørensen *et al.*, 2012 ▸) or on pattern recognition (Sørensen *et al.*, 2012 ▸), or involving a search of reflections and subsequent unit-cell optimization in 3D space (Wejdemann & Poulsen, 2016 ▸). Testing of these programs was performed on data sets artificially created by randomly rotating ‘grains’ with exactly defined unit-cell parameters, and there is no information on how well these programs would work with real data sets where one may need to consider statistical and instrumental errors in the positions of reflections in the reciprocal space, or deal with ‘junk’ reflections characteristic of the XRD data sets originating from high-pressure experiments in DACs. Another important problem is the long program running time; *e.g.* according to Wejdemann & Poulsen (2016 ▸), indexing of 500 cementite grains takes 5 days.

**Figure 1:**
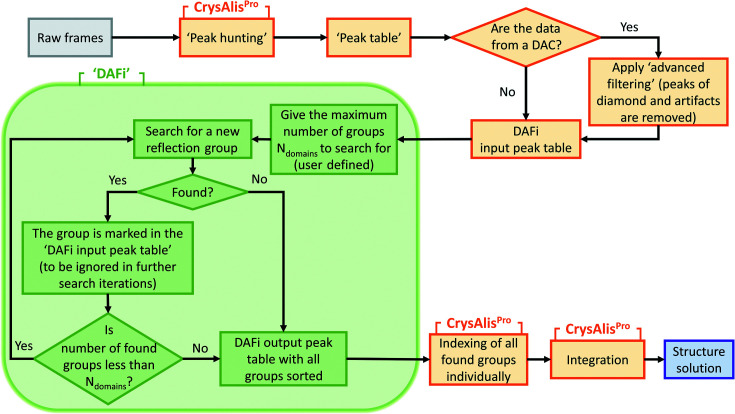
A data flowchart for the analysis of single-crystal XRD data from polycrystalline materials. The *DAFi* program is implemented to sort groups of reflections originating from individual single-crystal domains (see the text for detailed explanations).

In this article, we describe our methodological approach to the analysis of XRD data from polycrystalline materials and present the *DAFi* program which helps to automate the search for individual crystalline domains. A flowchart of the analysis is shown in Fig. 1 ▸. The *DAFi* program can be applied at that stage of the analysis when the diffraction from individual crystalline domains should be sorted. The algorithm does not need any *a priori* crystallographic knowledge, and there is no limitation on the phase composition of polycrystalline material and the number of crystalline domains of each phase. The algorithm is implemented with C++ code. Its important advantage is the extremely high speed of data processing. With the number of reflections in the input XRD data set (input peak table) equal to *N*_reflections_, the algorithm has 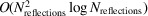 time complexity on a single-core processor, so that a typical computational time is several minutes. Implemented multithreading capability allows a further decrease of the computational time by dividing by the number of processor cores.

While the *DAFi* program enables the diffraction data of each domain to be separated from those of other domains, the data can be further processed using standard methods of single-crystal X-ray crystallography aimed at structure solution and refinement. The output of the current version of the *DAFi* program is compatible with the *CrysAlis^Pro^* software, which performs indexing of each found domain individually with just one click. However, there will not be a problem using the *DAFi* output file(s) with other standard indexing algorithms implemented in any available crystallographic programs. The algorithm of the *DAFi* program is described in detail below.

## 2. Algorithm

### 2.1. Input and output data

The algorithm requires only a set of coordinates of all reflections in the reciprocal space. Currently, the *DAFi* program reads these coordinates from the peaktable.tabbin file generated by the *CrysAlis^Pro^* software after ‘peak hunting’ (Fig. 1 ▸). If the XRD data originate from high-pressure experiments in a DAC, ‘advanced filtering’ (Koemets, 2020 ▸) is applied to eliminate the peaks produced by diamonds and the other diffraction artifacts associated with such a type of XRD raw data. After the ‘DAFi input peak table’ data processing, the *DAFi* program generates the output file(s), which is the ‘DAFi output peak table’ with the subsets of peaks sorted and numbered in the course of the search (see below for details). This means that the *DAFi* program updates the initial *CrysAlis^Pro^* peaktable.tabbin file by marking each reflection with the number of the subset (subset ID) to which it belongs.

### 2.2. General structure of the algorithm

The ‘peak table’ generated by the *CrysAlis^Pro^* software presents all diffraction peaks produced by a polycrystalline sample, which are visualized as a set of points in the reciprocal space. The whole set of points is a result of a superposition of numerous ‘subsets’ – the reciprocal-lattice points which belong to individual crystalline domains. Thus, if a subset is identified, then it can be indexed separately using standard crystallographic programs, and the crystal structure of the associated domain can be solved and refined.

Sorting subsets in the whole pattern of points in the reciprocal space is exactly the task of the *DAFi* program. The advantage of the implemented algorithm is that it selects the subsets purely geometrically, considering only a definition of a lattice (no time-consuming indexing is involved). As any 3D lattice is defined by three lattice vectors, the latter define three directions in a 3D space and the distances between the adjacent lattice points in these three directions. Obviously, a lattice can be recognized if considered as rows of equally distant points aligned in one direction, so the algorithm relies on finding such rows (*i.e.* a direction vector and a ‘proper’ distance between the adjacent points along the direction vector). This simplifies the search, which is realized iteratively. As soon as one subset of points is found, it is separated from the pool of all points, and only the remaining ones are considered in the next search.

**Figure 2:**
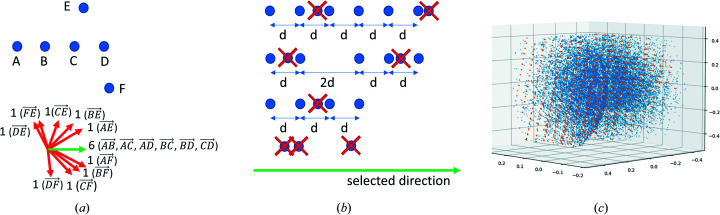
Illustration of the two main stages of the algorithm. (*a*) First stage: finding a set of possible directions (shown here by arrows) for a given set of reflections (here blue points A through F) and selecting the ‘best’ one(s) to consider at the second stage. Among the ten directions found for the set of points A, B, C, D, E, F, the ‘best’ one (shown by the green arrow) is identified as that corresponding to the largest number of collinear vectors. (*b*) The second stage: finding the ‘proper’ distance between the reflections (here denoted as ‘d’) in the selected direction. (*c*) An example of a view of the reciprocal space with the subset of points (orange dots) found in the initial set (blue dots).

The algorithm consists of two main stages:

(i) Finding a set of possible directions [Fig. 2 ▸(*a*)] and selecting the ‘best’ ones to consider at the second stage.

(ii) Finding the ‘proper’ distance between the reflections for a given direction [Fig. 2 ▸(*b*)] and identifying the nodes of the reciprocal lattice [Fig. 2 ▸(*c*)] generated by the chosen pair (direction, distance).

Combining these two parts we can find the ‘best’ pair (direction, distance), which corresponds to the biggest group of reflections belonging to one single-crystal domain. In Section 2.3 we describe different approaches to finding a set of possible directions, while in Section 2.4 we present an effective way to find the correct group of reflections for the given direction.

For the convenience of further mathematical description of the algorithm, the terms used below are defined as follows:

A *point* is a single diffraction reflection in the reciprocal space. The *points* are denoted as 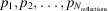 and are represented as radius vectors 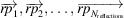 in a 3D space.

A *row* is a subset of reflections that lie on the same line in the reciprocal space.

A *group* is a subset of reflections in the reciprocal space belonging to a distinct single-crystal domain.

### 2.3. First stage of the algorithm

Before the main algorithm, *point* normalization is applied:

(i) All radius vectors are shifted by the vector 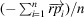 , which shifts the center of the *points*’ system to the coordinate 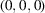 .

(ii) The coordinates 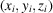 of each radius vector are divided by the maximum absolute value of the corresponding coordinate among all radius vectors (*i.e.* 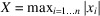 , 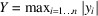 , 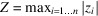 ). After that all radius vectors’ coordinates are transformed into 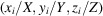 and belong to the range [−1; 1].

The shift described in the first step of the normalization procedure aims exclusively to improve the stability of the algorithm during the calculations. Although in practice the shift is very small, the shifting at the very beginning makes the algorithm more stable due to coordinates being transformed into a more uniform distribution. At the same time, the second part of the normalization procedure is important for further calculations [especially for the correct use of allowed absolute and relative errors (epsilon constants) in the second stage].

It is easy to see that the direction vector that determines the *group* will be equal to the direction vector between some two initial *points*. So, the most straightforward approach is to create a set of possible directions as a set of all direction vectors between each pair of initial *points*. However, such a set has a size of 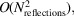 which is too large for the second stage of the algorithm. In Section 2.3.1 we propose a simple way to select only 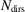 ‘best’ directions out of all 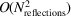 , where 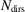 is any integer constant (naive approach), and in Section 2.3.2 we propose an improved version of such a selection (smart approach). Both naive and smart approaches are implemented in *DAFi* and the user can select which one to use in the configuration file.

#### 2.3.1. Naive approach

Ideally, we would like to select directions along which the second part of the algorithm will produce the largest possible *group*. We do not know in advance which directions are the ‘best’; however, we can see that if a *group* consists of *k* *rows* with sizes 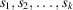 , then there are exactly 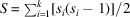 pairs of initial *points* that produce the same direction vector. This allows us to define the ‘best’ direction as the direction with a maximum number of pairs of initial *points* that produce it. However, because the initial *points* are real valued (have non-integer coordinates), all *S* generated vectors can differ slightly. To compare different real-valued vectors we transform them in two steps.

Before the first step of transforming a vector 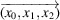 , where 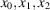 are the coordinates of the real-valued vector that we are transforming, index 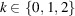 is found such that 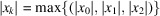 .

In the first step we make a transformation after which opposite vectors are considered to be equal: 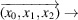 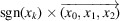 .

In the second step we transform the obtained vector to an integer-valued triplet: 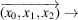 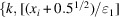 , 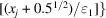 , where *i* and *j* are two indices from 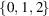 not equal to *k*, 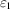 is a constant representing the allowed absolute error, and square brackets denote the integer part of a fractional number.

To illustrate this, let us consider a numerical example. Suppose that we want to transform a direction vector 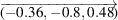 . 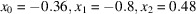 . Note that 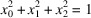 , because we are working with just a direction. Then the three steps of the transformation will be the following:

(i) 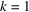 because 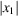 is maximum among 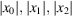 .

(ii) 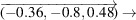 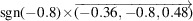 = 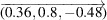 .

(iii) 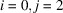 because there are only two indices from 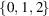 which are not equal to 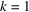 .

Let us also assume that 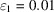 . Then we get the following integer-valued triplet:

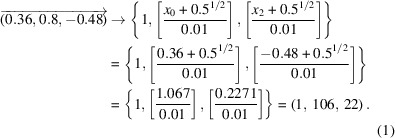




After such a transformation, each direction is represented as an integer-valued triplet with values in the range 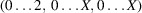 , where 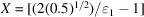 , so we can calculate a distribution of all directions using an array of size 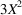 . After distribution calculation, we can find the top 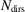 directions in time 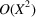 using a standard selection algorithm for finding the *k*th-order statistic (Blum *et al.*, 1973 ▸). To transform the integer-valued triplet back to the real-valued vector, we can use the following formula:

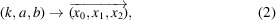

where 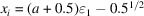 , 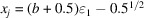 , 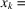 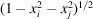 , and 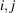 are two indices from 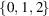 not equal to *k*.

This approach is the most straightforward way to select the ‘best’ 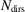 directions; however, it has drawbacks. The main one is that this approach does not use information about distances between *points*, which means that even with a large number of *points* lying on the same line, the second part of the algorithm may still not find the *group* if these *points* are located at unequal distances.

#### 2.3.2. Smart approach

Below we present the second approach to select the ‘best’ 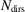 directions, which does not have the drawbacks mentioned above. We are still going to select the ‘best’ 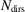 directions from some distribution; however, instead of creating a distribution from all 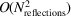 vectors, we will use only some of the more important of them. Namely, let us iterate over the ‘center’ *point* 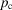 and find all possible *rows* of size at least 4 that go through the *point* 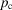 and consist of only equidistant *points*. In order to do this, first of all let us group all other 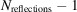 *points* in *rows* with respect to our center *point* 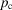 . This can be done by clustering all direction vectors 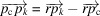 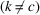 , similarly to the method described in Section 2.3.1. After this, for each *row*, we can independently find the largest subset of *points* where each *point* lies at an equivalent distance from the previous one. To do this, let us find out, for each *point* 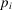 , at which distances *d* it will be in the same *row* as a *point* 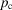 . Let us denote by *D* the distance between *points* 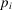 and 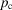 . Then we can say that 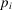 is the *k*th *point* in a *row* with 0th *point* 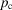 if the following holds: 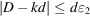 , where 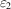 is some small constant that allows a small absolute error. From this inequality we can obtain that valid distances form the following range: 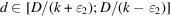 . After finding such ranges for all *points* 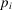 we can find the value of *d* that belongs to the largest number of ranges using the scanline algorithm (Klee, 1977 ▸). If this value is at least 3, then there exists a *row* that contains at least 4 *points* and with high probability belongs to a *group*. Only in such a case will we use the corresponding direction vector in our distribution. Such an approach takes 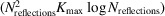 time, where 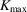 is the maximum *point*’s relative number on the *row* under consideration and 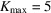 works well in practice.

**Figure 3:**
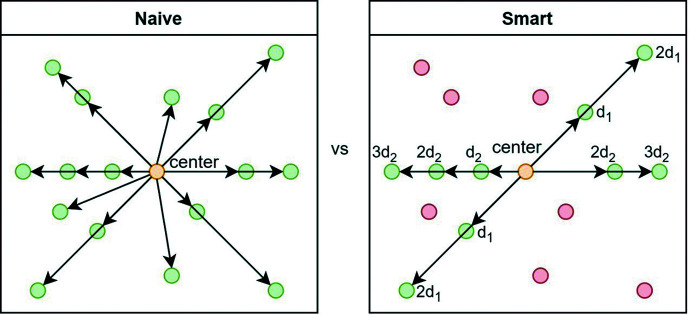
Comparison of naive and smart approaches. The naive approach implies consideration of all directions, while the smart one considers only the directions with *rows* of equidistant *points*.

The smart approach catches fewer ‘junk’ reflections (Fig. 3 ▸) and, therefore, provides a better distribution of direction vectors to the second stage of the algorithm. However, this approach is a bit slower, because instead of 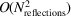 time, it requires 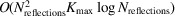 .

### 2.4. Second stage of the algorithm

Given a direction vector 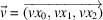 , we have to find the ‘best’ distance *d* between adjacent *points* towards a direction 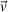 that generates the *group* of maximum size. Let 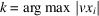 . Then we can project all initial *points* to a plane 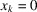 : radius vector 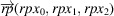 of *point* *p* will be transformed to 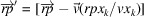 . After such a transformation, all *points* that belong to the same *row* in the direction 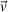 will be projected to the same point on a plane. This allows all different *rows* to be obtained by clustering of all projected *points*. Such clustering can be done in linear time using radix sort (Cormen *et al.*, 2001 ▸) and two linear passes that select equal *points* in 2 × 2 grid squares. After grouping all *points* into *rows*, we can create an array *ds* of all distances between adjacent *points* in the same *row* and choose *d* as the most frequent number in the array *ds*. Because all distances are real numbers, we have to use tolerance 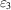 and choose *d* such that an interval 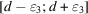 contains the most values from the array *ds*. Such a *d* can be found in linear time using the two pointers technique for maintaining a sliding window of size 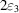 after sorting the array *ds*.

After finding the *d* value, we can find the exact *group* formed by a pair 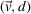 as a union of all largest valid subsets of *points* for each independent *row*. In order to find the largest valid subset for a given *row*, we introduce an auxiliary array ‘shifts’, where 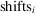 denotes the distance from a *point* 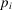 to the plane 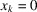 towards the direction 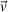 . Since the distance between all adjacent *points* in a *group*’s *row* is equal to *d*, for a valid subset of *points* it holds that all remainders 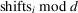 are equal, where mod denotes the modulo operation, *i.e.* 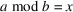 , 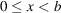 , 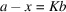 , 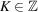 . This allows us to find the largest valid subset as the largest subset of *points* with equal values of 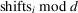 and pairwise different values of 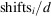 . It can be found in 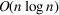 time using the two pointers technique for maintaining the set of all values 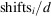 in a sliding window, where *n* is the number of *points* in the current *row*. Similarly to Section 2.3.2, the values 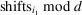 and 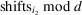 are considered equal iff 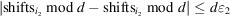 . The program has a configuration file that allows one to flexibly adjust all necessary parameters and in particular values 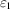 and 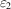 . Smaller values of tolerance will result in a more precise *group*; however, the found *group* will contain fewer reflections.

The time complexity of this stage can be estimated as 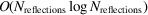 per direction, so the total time complexity for processing all best 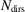 directions found in the previous stage is 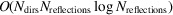 .

### 2.5. Speed optimizations

Without any optimizations, the program finds all *groups* one by one, so the total time complexity is

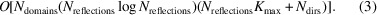

There are, however, some implemented optimizations that allow the algorithm to be significantly speeded up:

(i) Both stages of the algorithm allow the use of multithreading (in the first stage several threads uniformly process 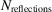 ‘center’ *points*, and in the second stage several threads uniformly process 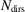 different best directions from the first stage).

(ii) The distribution of the ‘best’ directions is calculated only at the beginning of the program and, instead of recalculation from scratch on the following iterations, the distribution is just maintained by subtracting the impact of the removed *points* from the found *group* in time 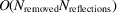 , where 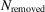 is the number of *points* in the last *group* found.

(iii) In fact, the algorithm finds 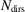 different *groups* in one iteration (one for each direction from the first stage), so there is an option to choose not just the largest *group*, but 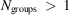 largest *groups* at once. This is done by firstly selecting the largest *group*, then the largest *group* with *points* not selected in the first *group*, and so on. Such an option allows the algorithm to be speeded up 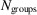 times; however, it may slightly decrease the quality of the search.

When combined, such optimizations allow the algorithm to be speeded up to the time complexity

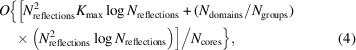

where 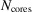 is the number of processor cores and 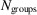 is the number of *groups* to be found in one iteration. Assuming that 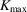 , 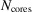 , 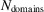 and 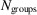 are all constants, the total time complexity can be simplified to 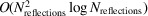 .

## 3. Examples of application

The testing of the *DAFi* program was performed on SC-XRD data sets obtained from real polycrystalline samples: (i) a natural basalt rock and (ii) a piece of yttrium (Y) embedded into molecular nitro­gen and laser-heated in a DAC. The results of these tests are described below as examples 1 and 2.

*Example 1. Study of a sample of basalt rock from the Rauher Kulm mountain/SC-XRD data collected using an in-house diffractometer.* Basalt rock is a natural polycrystalline aggregate of several minerals. A sample of basalt was collected by LD and ND at the Rauher Kulm mountain, which is a paleovolcano located in the Upper Palatinate region of the state of Bavaria, 23 km southeast of Bayreuth (Germany). A small isometric dark-gray grain of the rock (of about 40 µm in diameter) with sub-grains barely distinguishable under an optical microscope (×200) was mounted on a goniometer head. A single-crystal XRD data set was collected using a diffractometer equipped with a Bruker D8 platform (the three-axis goniometer), an APEX detector and an Ag *K*α Incoatec IµS source (beam size of ∼50 µm FWHM, half-sphere data collection, a collection time of 60 s with a step of 0.3°, 1265 frames). The peak hunting procedure in the *Crys­Alis^Pro^* software found 2928 reflections.

**Figure 4:**
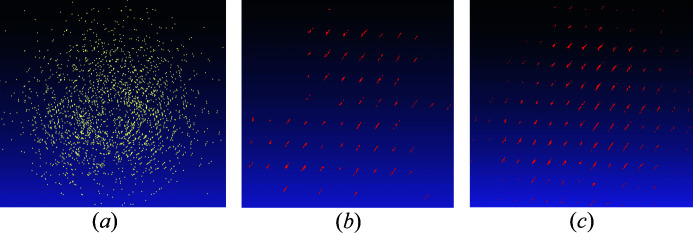
Reciprocal space representing SC-XRD data from a sample of basalt: (*a*) all reflections; (*b*) reflections of group 1 as found by the *DAFi* program; (*c*) reflections of group 1 extended by *CrysAlis^Pro^* software.

The search for 18 groups of reflections (the number set by the user) in a pool of 2928 reflections took the *DAFi* program 31 s (Fig. 4 ▸ and Table 1 ▸). Each of the 18 groups found had its own size (the number of reflections included in the group). In the course of further data processing and indexing using *CrysAlis^Pro^*, some groups were merged, as the *CrysAlis^Pro^* program recognized them as related to the same single-crystal domain. For example, eight groups (3, 5, 6, 9, 10, 14, 16 and 18) were merged with group 1, whose size increased from 421 (as found by *DAFi*) to 1312 reflections after indexing (see Table 1 ▸). Further processing in *CrysAlis^Pro^* revealed crystallographic parameters of the mineral olivine. The olivine crystallite is mosaic, and its nine slightly misaligned domains were recognized by *DAFi* separately, whereas *CrysAlis^Pro^*, due to the higher tolerance (0.125 in this particular case), counted the whole crystallite as one domain. Thus, *CrysAlis^Pro^* revealed the crystallographic data for seven independent single-crystal domains of three different minerals: three domains of phlogopite, three of chromite and one domain of olivine (Table 1 ▸).

*Example 2. Study of products of the reaction of yttrium and nitro­gen in a double-sided laser-heated DAC at 50 GPa.* A piece of yttrium was placed in the sample chamber of a BX90-type large X-ray aperture DAC (Kantor *et al.*, 2012 ▸) equipped with Boehler–Almax-type diamonds with 250 µm culets. Molecular nitro­gen was then loaded into the DAC using a high-pressure gas loading system. The sample was compressed to ∼50 GPa and laser-heated (λ = 1064 nm) to 2000 (200) K using the double-sided laser heating system operating at the P02.2 beamline at the PETRA III synchrotron. A single-crystal data set was collected at the same P02.2 beamline (λ = 0.2908 Å, beam size 1.8 × 2 µm FWHM, acquisition time 4 s, angular ω step 0.5^o^, 132 frames). See Aslandukov *et al.* (2021 ▸) for more experimental details.

The peak hunting procedure in *CrysAlis^Pro^* found 68 846 reflections. Since this high-pressure experiment was conducted in a DAC, a lot of undesired reflections from diamonds, the pressure-transmitting medium, the material of the gasket and other artifacts were present in the data set. Therefore, a procedure of ‘advanced filtering’ was applied to remove such reflections before the execution of the *DAFi* program. To realize such a ‘clean-up’, a special script was written by E. Koemets and M. Bykov, and then incorporated into the *CrysAlis^Pro^* software. After the filtering, 44 312 reflections were left out of 68 846. The size of the *DAFi* input data set (‘DAFi input peak table’) was still huge. In such cases, it is more reasonable to search for several strongly diffracting domains of different phases than for all single-crystal domains. A search for ten groups in 44 312 reflections took the *DAFi* program 5 min 25 s.

**Figure 5:**
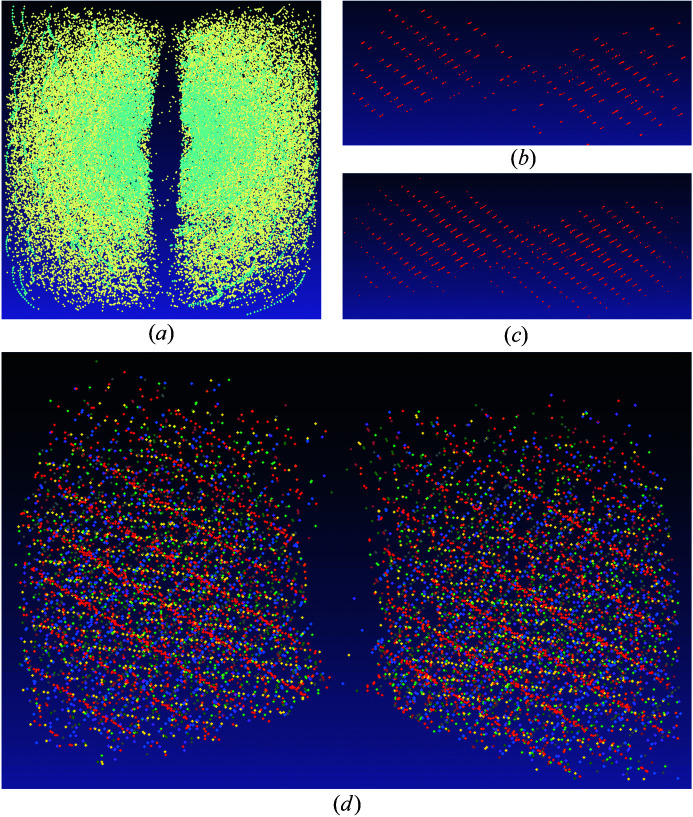
Reciprocal space representing SC-XRD data from a sample of Y+N_2_ in a DAC: (*a*) all reflections (cyan reflections are those filtered after applying ‘advanced filtering’); (*b*) reflections of group 1 belonging to the first Y_5_N_14_ domain found by the *DAFi* program; (*c*) reflections of group 1 belonging to the Y_5_N_14_ domain extended by *CrysAlis^Pro^*; (*d*) reflections of ten groups (1 through 10) belonging to ten Y_5_N_14_ domains marked by ten different colors.

The results of the search are shown in Fig. 5 ▸ and Table 2 ▸. It appeared that all ten groups of reflections belong to the same phase. Each group was indexed independently in *CrysAlis^Pro^* [see Table 2 ▸ and Figs. 5 ▸(*b*) and 5 ▸(*c*) as an example], and the crystal structure of the phase (identified as Y_5_N_14_) was solved and refined for each of its single-crystal domains (Aslandukov *et al.*, 2021 ▸) (*e.g.* for domain 6, the integration led to *R*_int_ = 6.47%; based on 597 independent reflections, the structure of Y_5_N_14_ was solved and refined to *R*_1_ = 4.88%). Note that the unusual stoichiometry of the Y_5_N_14_ phase was not known initially and was determined as a result of the crystal structure solution and refinement using the standard crystallographic software *OLEX2* (Dolomanov *et al.*, 2009 ▸), considering that the elements present in the system were known. In the example of Y_5_N_14_, only a piece of yttrium and nitro­gen were loaded into the DAC, thus limiting the set of possible elements (Y, N) in the new compound. Other possible elements (for example, C from the diamond anvils, Re from the gasket or other impurities in the initial sample) would have to be taken into consideration in the case of unsatisfactory structure refinement (which was not the case for Y_5_N_14_).

The *DAFi* program could have been run to find more domains. However, this would have made sense only if there were other phases in the sample. In this particular case, a quick check of the powder diffraction pattern generated for the whole data set showed no extra reflections apart from the found phase; therefore there was no reason to continue the search.

## 4. Summary

Existing indexing algorithms for single-crystal data analysis implemented in available crystallographic programs have no proven record of application to SC-XRD data processing from a multiphase mixture of microcrystalline samples. Superposition of numerous reflections originating from a large number of single-crystal domains of the same and/or different (especially unknown) phases precludes the sorting of reflections coming from individual domains, making their automatic indexing impossible. The *DAFi* algorithm presented in this work is designed for a quick search for subsets of reflections from individual domains in a whole set of SC-XRD data from a seemingly polycrystalline sample. Further indexing of all found subsets can be easily performed in one click using widely accessible crystallographic packages such as *CrysAlis^Pro^*. The fact that the algorithm presented above neither requires *a priori* crystallographic information nor is limited by the number of the various phases and their individual domains makes *DAFi* a powerful software tool to be used for studies of multiphase polycrystalline and microcrystalline (powder) materials. It has been shown to be especially valuable for the analysis of single-crystal diffraction data from products of chemical reactions being realized in laser-heated DACs. Such data are always very complex due to (i) the presence of undesired reflections from diamond anvils and gaskets, and other technical and diffraction artifacts (*e.g.* ‘bad’ or ‘saturated’ detector pixels, or reflections from the body of the DAC itself), and (ii) the limited opening angle of DACs, which shadows a part of the Ewald sphere. To our knowledge, there are no existing software tools capable of finding the domains of unknown phases in such a complicated XRD data set as in example 2. The *DAFi* program tackles the task within a few minutes and finds several strongly diffracting domains, so that their XRD patterns can be indexed, the data integrated, and the crystal structures solved and refined. The high performance of the proposed algorithm allows the use of this program for online processing of the XRD data directly during experiments at synchrotron facilities.

The *DAFi* program is not designed to be effective with non-merohedral twins, where a large fraction of reflections are overlapped, while some of them overlap only partially or do not overlap. Once *DAFi* finds the reflection group, the program removes it from consideration for the next iterations. If reflections do not overlap, *DAFi* finds two separate reflection groups which can be processed afterwards by the user. If reflections overlap partially, *DAFi* finds two separate reflection groups; however, the first group would contain all reflections of the first crystal in a twin, while the second group would contain only non-overlapped reflections of the second one. If a large number of reflections overlap, the second group most likely will not be found.

The current version of the *DAFi* program does not find all reflections belonging to a particular single-crystal domain, as the algorithm searches for rows of at least three reflections along a certain direction, so that single reflections or those which are only two in a row are overlooked. Also, several groups of reflections can be found to belong to the same domain, as in the 3D reciprocal space the algorithm searches for rows of reflections in only one direction. These technical peculiarities of the program are not crucial for further data processing, as the input and output format of the *DAFi* program are compatible with the *CrysAlis^Pro^* software. Moreover, the input and output formats of the *DAFi* program could be adapted to users’ needs and made to be compatible with other crystallographic software.

## 5. Distribution

The *DAFi* program and its documentation can be downloaded from https://github.com/AsMaNick/Domain-Auto-Finder/.

## Figures and Tables

**Table 1:** Results of the *DAFi* run on the data set collected from a sample of basalt

			Lattice parameters						
Group No.	Size of group found by *DAFi*	Size of group after indexing in *CrysAlis^Pro^* †	*a* (Å)	*b* (Å)	*c* (Å)	α (°)	β (°)	γ (°)	Minerals
1	421	1312	4.7881 (7)	6.0329 (10)	10.3079 (2)	90	90	90	Olivine
2	197	494	5.2989 (17)	8.911 (3)	9.755 (3)	90	105.47 (3)	90	Phlogopite
3	171	Merged with group 1							
4	137	372	8.425 (3)	8.425 (3)	8.425 (3)	90	90	90	Chromite
5	107	Merged with group 1							
6	57	Merged with group 1							
7	82	Merged with group 2							
8	57	Merged with group 3							
9	38	Merged with group 1							
10	41	Merged with group 1							
11	25	140	8.425 (3)	8.425 (3)	8.425 (3)	90	90	90	Chromite
12	28	Merged with group 3							
13	15	98	5.317 (8)	8.907 (5)	9.723 (9)	90	105.35 (12)	90	Phlogopite
14	16	Merged with group 1							
15	21	86	5.286 (9)	8.970 (17)	9.752 (19)	90	106.0 (2)	90	Phlogopite
16	20	Merged with group 1							
17	20	85	8.398 (6)	8.398 (6)	8.398 (6)	90	90	90	Chromite
18	26	Merged with group 1							

† Indexing performed with a tolerance of 0.125.

**Table 2:** Results of the *DAFi* run on the data set collected from a sample of Y+N_2_ in a DAC at 50 GPa

			Lattice parameters						
Group No.	Size of group found by *DAFi*	Size of group after indexing in *CrysAlis^Pro^* †	*a* (Å)	*b* (Å)	*c* (Å)	α (°)	β (°)	γ (°)	Phase
1	617	1286	8.4595 (3)	8.4595 (3)	4.7032 (5)	90	90	90	Y_5_N_14_
2	590	1044	8.4788 (6)	8.4788 (6)	4.6883 (3)	90	90	90	Y_5_N_14_
3	499	954	8.4527 (8)	8.4527 (8)	4.7034 (11)	90	90	90	Y_5_N_14_
4	485	997	8.4459 (5)	8.4459 (5)	4.7182 (7)	90	90	90	Y_5_N_14_
5	531	1199	8.4538 (4)	8.4538 (4)	4.711 (2)	90	90	90	Y_5_N_14_
6	483	1095	8.4737 (5)	8.4737 (5)	4.6922 (4)	90	90	90	Y_5_N_14_
7	478	1011	8.4710 (5)	8.4710 (5)	4.6977 (4)	90	90	90	Y_5_N_14_
8	375	984	8.4690 (5)	8.4690 (5)	4.7047 (19)	90	90	90	Y_5_N_14_
9	361	823	8.4606 (5)	8.4606 (5)	4.7065 (3)	90	90	90	Y_5_N_14_
10	308	935	8.4677 (9)	8.4677 (9)	4.699 (3)	90	90	90	Y_5_N_14_

† Indexing performed with a tolerance of 0.05.
